# Effects of Word Frequency, Word Length, and Visual Complexity on Chinese Sentence Oral Reading: An Eye Movement Comparison Study Between Children and Adults

**DOI:** 10.3390/jemr19030052

**Published:** 2026-05-13

**Authors:** Kunyu Lian, Junhui Pei, Feifei Liang, Jie Ma, Rong Lian, Xuejun Bai

**Affiliations:** 1 School of Health Sciences, Block A, Science and Technology Park, Fujian Medical University, No. 1 Xueyuan Road, Shangjie Town, Minhou County, Fuzhou 350122, China; 2 Faculty of Psychology, Tianjin Normal University, No. 393 Binshui West Road, Xiqing District, Tianjin 300387, China; 3 School of Psychology, Fujian Normal University, Qishan Campus, Fuzhou 350108, China

**Keywords:** oral reading, eye movements, word frequency, word length, visual complexity, developmental differences

## Abstract

This study investigated how word frequency, word length and visual complexity affect lexical processing during Chinese sentence oral reading, and whether these effects differ between developing and skilled readers. Third-grade children and adults read sentences aloud while their eye movements were recorded with an EyeLink 1000 Plus eye-tracker. Linear mixed-effects models revealed three main findings. First, children showed larger word-frequency and visual-complexity effects than adults, indicating less efficient lexical processing in developing readers. Second, word length moderated the effects of word frequency and visual complexity. Frequency effects were amplified for two-character words, whereas visual-complexity effects were stronger for single-character words on early measures and followed a different pattern on some late measures. Third, at the sentence level, children exhibited shorter forward saccades, more regressions and longer total reading times than adults. These findings provide developmental evidence for the visual and linguistic constraints hypothesis and show how visual recognition and overt phonological output jointly shape foveal lexical processing in Chinese oral reading.

## 1. Introduction

### 1.1. Research Area

Oral reading is a fundamental literacy skill that demands the rapid coordination of visual word recognition, lexical access, and phonological output. Unlike silent reading, oral reading requires that each word be explicitly decoded and pronounced, placing substantial demands on the cognitive system [1]. The study of eye movements during oral reading offers a uniquely informative window into these processes: because articulation necessarily lags behind visual encoding, the eyes typically lead the voice by several words, creating an “eye–voice span” that reveals the temporal dynamics of lexical processing under high cognitive load [2]. For children in the early stages of reading development, oral reading is not merely an experimental task but a daily educational practice that bridges the transition from oral to literate language skills [3,4]. Understanding how children and adults differ in their lexical processing during oral reading is therefore essential for both theories of reading development and instructional practice.

### 1.2. Research Problem

Word length, word frequency, and visual complexity are three fundamental lexical properties that jointly influence eye movement behaviour during reading. Word length effects have been documented across all writing systems studied [5,6,7], reflecting the decline in visual acuity from fovea to parafovea. Word frequency effects—the finding that low-frequency words receive longer fixations—are equally robust across alphabetic and logographic scripts [8,9,10]. Visual complexity, operationalized as stroke count in Chinese, exerts independent effects on early visual processing and lexical recognition [11,12]. However, no study has systematically examined how word length modulates word frequency and visual complexity effects during Chinese oral reading, and the developmental trajectory of these interactions remains unknown.

### 1.3. Research Objective

This study examines how word frequency, word length and visual complexity jointly influence eye movements during Chinese sentence oral reading in third-grade children and university adults. The main objective is to determine whether word length modulates the effects of word frequency and visual complexity, and whether these interactions differ as reading skill develops.

### 1.4. Research Aim and Questions

The research questions are as follows:

RQ1: How do word frequency, word length, and visual complexity interact to influence eye movement behaviour during Chinese sentence oral reading?

RQ2: How do these interactions differ between developing readers (third-grade children) and mature readers (university adults)?

We hypothesized that: (1) All three lexical properties would produce significant main effects on eye movement measures, with longer fixation times for low-frequency, long, and high-visual-complexity words. (2) Children would show larger word frequency and visual complexity effects than adults, reflecting less efficient lexical processing. (3) Word length would modulate the effects of word frequency and visual complexity, consistent with the visual and linguistic constraints hypothesis [13].

## 2. Background

### 2.1. Word Length Effects in Reading

Word length is one of the most robust predictors of eye movement behaviour during reading. Across alphabetic writing systems, shorter words are more likely to be skipped, receive fewer fixations, and elicit shorter gaze durations than longer words [5,6,14]. Both the E-Z Reader model [15] and the SWIFT model [16] attribute the word length effect to the decline in visual acuity from the fovea to the parafovea. As word length increases, a greater proportion of the word falls into the parafoveal region, where perceptual resolution is coarser, thereby reducing identification efficiency and necessitating longer fixations. Importantly, McDonald [17] demonstrated that six-letter and eight-letter English words matched for physical width still produced reliable differences in fixation duration, indicating that the effect is driven by the number of orthographic units rather than retinal extent alone. Hautala et al. [18] further showed that font type modulates the word length effect.

In Chinese—a script without interword spaces in which the vast majority of words consist of one or two characters—word length remains a critical determinant of eye movement control. The weight of evidence now favors flexible, word-based targeting strategies over fixed saccade length strategies [19,20,21]. Zang et al. [22] systematically compared single-character, two-character, and three-character target words and found that longer words elicited longer fixation durations, shorter saccade lengths, and lower skipping rates. Tong et al. [23] provided additional evidence that saccade amplitude varied with word length, supporting a word-centered saccade length hypothesis. The word length effect also shows developmental variation: older adults exhibit a smaller perceptual span during Chinese reading [24], and word length interacts with age on fixation duration [25]. Liang et al. [13] found that word length effects for newly learned words diminished with repeated exposure, suggesting that word length can operate at higher levels of lexical processing.

### 2.2. Word Frequency Effects in Reading

The word frequency effect—the finding that high-frequency words receive shorter fixations and are skipped more often than low-frequency words—has been documented in virtually every writing system studied [8,9,10]. In Chinese, readers spend less time fixating high-frequency words than low-frequency words across a range of measures [10,26]. Beyond its main effect on fixation duration, word frequency also influences the spatial parameters of eye movements: high-frequency words elicit longer forward saccades than low-frequency words [27,28,29]. Liu et al. [28] demonstrated that this saccade length effect is constrained by the availability of parafoveal preview.

Word frequency also interacts with other lexical properties. In Chinese, word frequency and visual complexity produce additive and interactive effects [30,31,32]. Yeh and Liu [33] confirmed an interaction between word frequency and visual complexity using tachistoscopic presentation, indicating that the ease of visual pattern recognition modulates lexical accessibility. Recent research has further shown that word frequency effects are shaped by task demands and reader characteristics: Wang et al. [34] reported that word frequency effects appeared earlier in silent reading (first fixation duration) than in oral reading (gaze duration), suggesting that phonological demands delay the manifestation of frequency effects. Zhang et al. [35] showed that fast readers were unaffected by word frequency, whereas slow readers exhibited substantially longer fixations on low-frequency words, supporting the lexical quality hypothesis.

### 2.3. Visual Complexity Effects in Reading

Visual complexity refers to the structural intricacy of a word’s visual form. In alphabetic scripts, visual complexity is confounded with word length, creating a methodological impasse for researchers who wish to isolate its effect [11]. Chinese offers a unique solution: because all characters occupy the same physical width, visual complexity can be manipulated independently of word length by varying stroke count [36]. Yang and McConkie [26] demonstrated that increasing stroke count significantly prolongs recognition time. Zhang et al. [12] found significant main effects of visual complexity on first fixation duration, gaze duration, and skipping rate during search reading. Liang et al. [13] showed that visual complexity effects interact with word length during novel vocabulary acquisition. Despite these converging findings, visual complexity has more often been treated as a nuisance variable, representing a significant gap that the present study addresses.

### 2.4. Oral Reading: Distinctive Characteristics

Oral reading presents a distinct cognitive profile that merits separate theoretical attention. Oral reading is universally slower than silent reading and produces more complex eye movement patterns, including longer fixation durations and more regressions [37,38]. These differences arise because oral reading requires allocation of cognitive resources to phonological encoding, articulatory planning, and prosodic control beyond those involved in visual word recognition [39].

A defining feature is the eye–voice span—the distance between the word currently being fixated and the word currently being articulated [2]. This temporal dissociation restricts the reader’s ability to speed up processing through strategic control. Research comparing silent and oral reading has revealed modality-specific patterns: Zang et al. [40] found that the benefit of a larger perceptual window was more pronounced in silent reading. Zhang et al. [41] demonstrated that low-frequency and high-visual-complexity words elicited the longest fixation durations in oral reading. Wang et al. [34] found that word frequency effects emerged earlier in silent reading than in oral reading, and Jia et al. [42] extended this finding to first-grade children.

The developmental dimension is particularly salient. Third grade represents a critical juncture: children have developed basic decoding skills but have not yet achieved the automaticity characteristic of adult reading [3,4]. These developmental limitations predict that children should exhibit larger effects of lexical variables than adults during oral reading, because less familiar and more visually complex words place disproportionate demands on processing resources already strained by overt articulation.

## 3. Materials and Methods

Eye movements were recorded using an EyeLink 1000 Plus eye-tracking system (SR Research Ltd., Ottawa, ON, Canada) with a sampling rate of 1000 Hz. Stimuli were presented on a 19-inch Dell monitor (75 Hz refresh rate, 1024 × 768 pixels resolution). Participants were seated 65 cm from the monitor with their head stabilized using a chin rest and forehead support. Stimuli were displayed in Song (SimSun) 26-point font, with each Chinese character subtending approximately 37 × 37 pixels (1.16 degrees of visual angle horizontally). Testing was conducted in a dimly lit, sound-attenuated room.

A total of 274 participants were recruited, comprising 137 adults (108 females, 29 males; mean age = 18.70 years, SD = 0.85, range = 18–22 years) and 137 third-grade children (61 females, 76 males; mean age = 9.89 years, SD = 0.42, range = 9–11 years). Third grade was selected because it represents a critical developmental period for oral reading fluency in Chinese children [3,4]. All participants were native Mandarin Chinese speakers with normal or corrected-to-normal vision. None reported any history of reading or neurological disorders.

The experiment employed a 2 (Group: adults vs. children) × 2 (Word Frequency: high vs. low) × 2 (Word Length: single-character vs. two-character) × 2 (Visual Complexity: complex vs. simple) mixed factorial design. Group was a between-subjects factor; Word Frequency, Word Length, and Visual Complexity were within-subjects factors. Dependent variables comprised three word-level eye movement measures (first fixation duration, gaze duration, and total fixation duration) and three sentence-level measures (forward saccade size, regression count, and total reading time).

Participants were tested individually. A three-point horizontal calibration was performed and repeated until the average calibration error was below 0.30 degrees of visual angle. A validation check was conducted before each experimental block, with recalibration performed when validation error exceeded the criterion. Following successful calibration, participants were instructed to read each sentence aloud naturally and accurately. In approximately 25% of trials, a yes/no comprehension question appeared after the sentence to ensure attention to meaning. The session began with six practice trials, followed by 176 experimental trials presented in randomized order across four blocks of 44 sentences each, with a 10-min break between blocks. Each trial began with a drift-correction dot; once fixated, the experimenter initiated the trial and the sentence appeared. Participants pressed the spacebar after oral reading. The entire session lasted approximately 120 min.

All eye movement data were analysed using linear mixed-effects models (LMMs) in the lme4 package [43] within the R environment [44]. LMMs allow simultaneous modelling of participant and item variability as crossed random effects [45]. We adopted a maximal random effects structure including random intercepts for participants and items, with random slopes for within-subjects factors where convergence permitted. Binary outcome measures were analysed using generalized linear mixed-effects models with a logistic link function. Continuous dependent variables were log-transformed where appropriate. All *p* values for LMM analyses were derived from the asymptotic t distribution for linear models and from the standard normal (z) distribution for generalized linear models. The significance level was set at alpha = 0.05. Effect sizes are reported as unstandardized regression coefficients (b) with standard errors (SEs).

## 4. Ethics Statement

The study was conducted in accordance with the Declaration of Helsinki and approved by the Institutional Review Board of Biomedical Research, Fujian Medical University (protocol code FYIRB No. 259 (2024) and date of approval [8 June 2024]). Informed consent was obtained from all adult participants and from the parents or legal guardians of all child participants prior to data collection.

## 5. Stimulus Materials

Sentences were segmented into words using the Contemporary Chinese Dictionary and the SUBTLEX-CH word frequency corpus [46]. Words present in either the dictionary or the corpus were identified as individual word units. After removing the first and last words of each sentence, a total of 2375 words were segmented. The segmented words were ranked by percentile on word frequency and stroke count, respectively. Words in the top 27th percentile were classified as the high group, and words in the bottom 27th percentile were classified as the low group. The number of words in each group is shown in Table 1.

The mean word frequency was 8901 per million (SD = 9035) for the high-frequency group and 141 per million (SD = 175) for the low-frequency group. An independent-samples *t*-test revealed that word frequency was significantly higher in the high-frequency group than in the low-frequency group, t(525) = 22.23, *p* < 0.001. The mean number of strokes was 21.2 (SD = 2.86) for the high visual complexity group and 6.26 (SD = 2.19) for the low visual complexity group. An independent-samples *t*-test revealed that stroke count was significantly higher in the high visual complexity group than in the low visual complexity group, t(474) = 83.34, *p* < 0.001.

Examples of the experimental materials are presented below (vertical bars did not appear between words in the actual experiment). The word 懂得 (dǒng de, “understand”) belongs to the high-frequency, high-visual-complexity group; 他 (tā, “he”) belongs to the high-frequency, low-visual-complexity group; 其实 (qí shí, “actually”) belongs to the low-frequency, high-visual-complexity group; and 长大 (zhǎng dà, “grow up”) belongs to the low-frequency, low-visual-complexity group.

Example sentence: 已经|长大|了|的|王子|懂得|国王|其实|是|为|他|好。

The experimental materials consisted of 176 simple Chinese sentences adapted from third-grade Chinese language textbooks. Each sentence contained between 12 and 18 Chinese characters (M = 14.6 characters). Sentence difficulty was assessed by 15 elementary school students who did not participate in the formal experiment, using a 5-point rating scale; the mean difficulty rating was 4.73 (SD = 0.35). Sentence fluency was independently rated by 15 adults (M = 4.29, SD = 0.42).

All sentences were segmented into words using the SUBTLEX-CH Chinese word and character frequency corpus [46] and the Modern Chinese Dictionary as segmentation standards. After removing the first and last words of each sentence, a total of 2375 word tokens were identified, yielding 732 unique word types (157 single-character words, 561 two-character words, 13 three-character words, and 1 four-character word). Three- and four-character words (1.91% of the total pool) were excluded to ensure sufficient power for the factorial design. Word frequency was dichotomized into high-frequency and low-frequency categories based on SUBTLEX-CH norms. Visual complexity was operationalized as the number of strokes per character and dichotomized into high-complexity and low-complexity conditions. For two-character words, visual complexity was determined by the mean stroke count of the constituent characters. An example sentence is: 陈老师说过可以运用乘法解决这道数学题。 (“Teacher Chen said that multiplication can be used to solve this math problem”).

Visual complexity was operationalized as the number of strokes per character. For single-character words, visual complexity was determined by the stroke count of that character. For two-character words, visual complexity was computed by averaging the stroke counts of the two constituent characters; for instance, a word composed of characters with 10 and 6 strokes would receive a visual complexity value of 8 (the mean of the two stroke counts).

## 6. Results

All data were analysed using linear mixed-effects models (LMMs) implemented in the lme4 package [43] within the R environment [44]. Compared with conventional ANOVA-based approaches, this analytical method offers several advantages. First, traditional methods require separate examination of subject- and item-level variability; however, when participant analysis and item analysis yield inconsistent results, it becomes difficult to draw definitive conclusions. The use of mixed-effects models circumvents this issue by adopting a maximal random effects structure [45], wherein participants and items are specified as crossed random effects within a single model, thereby avoiding discrepancies between participant- and item-based analyses. Second, conventional methods aggregate data by computing mean values for each participant or item on a given measure, resulting in substantial loss of information. In contrast, mixed-effects models incorporate all raw observations into the analysis, thereby maximising the statistical utility of the data.

In the whole-sentence analysis, group was included as a fixed factor in the model. In the word-level local analyses, group, word frequency, visual complexity, and their interactions were entered as fixed factors.

In Figure 1, Figure 2, Figure 3, Figure 4, Figure 5, Figure 6, Figure 7 and Figure 8, the horizontal axes represent standardized scores. This transformation removes the original units of measurement and allows variables with different scales to be compared more directly.

### 6.1. Comprehension Accuracy

All participants demonstrated adequate comprehension, with overall accuracy rates exceeding 80%. University students achieved a mean comprehension accuracy of 94%, whereas children achieved 88%. An independent-samples *t*-test revealed that adults’ comprehension accuracy was significantly higher than that of children, t(261) = 7.11, *p* < 0.001. This confirms that both groups were engaged in meaningful reading during the oral reading task.

### 6.2. Data Screening

Prior to analysis, eye movement data were screened according to established criteria [6,7]. Fixations with durations shorter than 80 ms or longer than 1200 ms were excluded. Trials were excluded if (1) eye-tracking data were lost due to track loss (0.03% of all trials), (2) a sentence received fewer than five fixations (0.66%), or (3) the data point exceeded three standard deviations from the cell mean (0.51%). In total, 1.2% of the data were removed.

### 6.3. Sentence-Level Analysis

Following previous research on Chinese reading [47,48,49], we analysed eye movement patterns at the sentence level. Three measures were computed: forward saccade size (mean amplitude of forward-directed saccades, in characters), regression count (total number of regressive saccades), and total reading time (sum of all fixation durations, in milliseconds). Descriptive statistics are presented in Table 2, and fixed-effect estimates in Table 3.

Forward Saccade Size. The main effect of Group was significant (b = −0.22, SE = 0.02, t(271) = −9.23, *p* < 0.001). Elementary students produced significantly shorter forward saccades (M = 1.70 characters, SD = 0.53) than adults (M = 2.11, SD = 0.62).

Regression Count. The main effect of Group was significant (b = 0.53, SE = 0.05, t(270) = 11.21, *p* < 0.001). Elementary students made significantly more regressive saccades (M = 6.46, SD = 3.61) than adults (M = 3.80, SD = 2.12).

Total Reading Time. The main effect of Group was significant (b = 0.58, SE = 0.02, t(253) = 24.09, *p* < 0.001). Elementary students exhibited substantially longer total reading times (M = 8876 ms, SD = 2881) than adults (M = 4877 ms, SD = 1153).

In practical terms, children required approximately 82% more time than adults to read a sentence, highlighting the substantial developmental gap in oral reading efficiency.

Taken together, the sentence-level analyses revealed robust group differences, indicating that children demonstrated less efficient oral reading, as reflected in shorter forward saccades, more regressions and longer total reading times.

### 6.4. Word-Level Analysis

To examine the effects of word frequency, word length, and visual complexity on lexical processing during oral reading, we conducted word-level analyses on three eye-movement measures. Linear mixed-effects models (LMMs) were fitted for all measures.

The measures analysed were as follows: (1) first fixation duration, defined as the duration of the first fixation during first-pass reading and taken to reflect early lexical access; (2) gaze duration, defined as the time from the first fixation on a word until gaze first exits that region and taken to reflect lexical identification; and (3) total fixation duration, defined as the sum of all fixations on the target word and taken to reflect later processing.

Table 4 presents the fixed-effects results for first fixation duration, gaze duration, and total fixation duration.

#### 6.4.1. First Fixation Duration

First fixation duration, indexing the earliest stage of lexical access, revealed significant main effects of group, word length, and visual complexity, but no main effect of word frequency.

Main effects. Children exhibited significantly longer first fixation durations than adults (b = 0.09, SE < 0.01, t(45,930) = 15.00, *p* < 0.001). Long words received longer first fixations than short words (b = −0.04, SE < 0.01, t(4453) = −6.28, *p* < 0.001). Word frequency did not significantly affect first fixation duration (b < −0.01, SE < 0.01, t(34,850) = −0.97, *p* = 0.330). High visual complexity words elicited longer first fixations than low visual complexity words (b = 0.05, SE < 0.01, t(28,990) = 8.40, *p* < 0.001).

**Figure 1:**
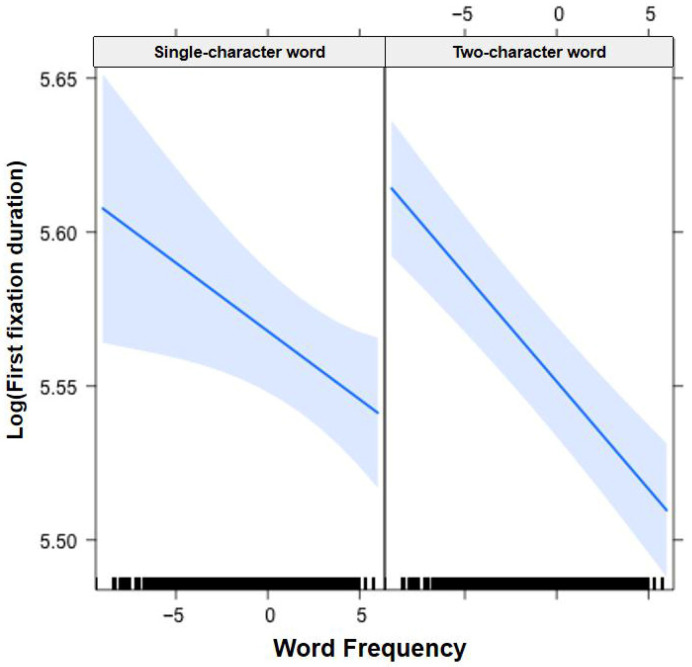
Word Length × Word Frequency interaction on first fixation duration. All participants showed a larger word frequency effect for two-character (long) words than for single-character (short) words. Note. The x-axis represents standardized log-transformed word frequency, and the y-axis represents the natural logarithm of first fixation duration. The left panel shows results for single-character words, and the right panel shows results for two-character words. Blue solid lines indicate linear regression fit lines, and light blue shaded areas represent 95% confidence intervals around the fit lines. Black ticks at the bottom are rug plots showing the distribution of observations along the x-axis.

**Figure 2:**
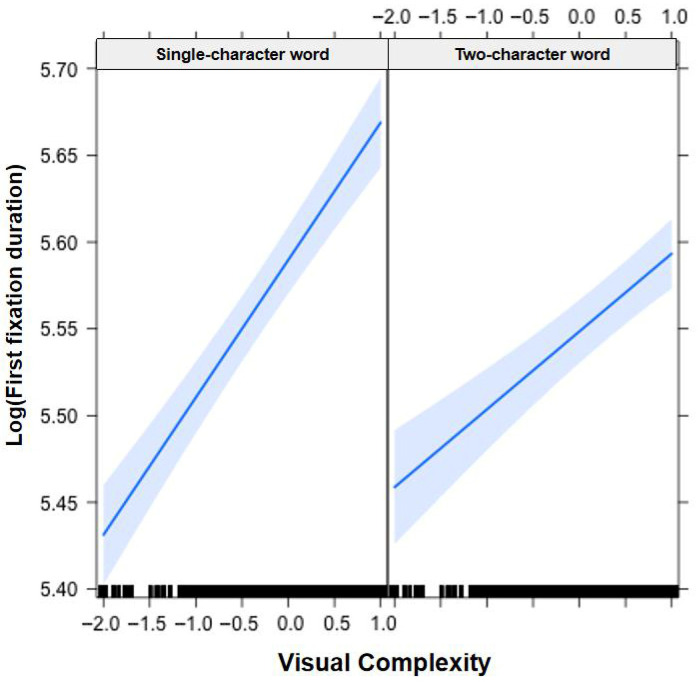
Word Length × Visual Complexity interaction on first fixation duration. All participants showed a larger visual complexity effect for single-character (short) words than for two-character (long) words. Blue solid lines indicate linear regression fit lines, and light blue shaded areas represent 95% confidence intervals around the fit lines. Black ticks at the bottom are rug plots showing the distribution of observations along the x-axis.

Interactions. The Group × Word Frequency interaction was not significant (b < −0.01, SE < 0.01, t(58,440) = −0.69, *p* = 0.489), nor was the Group × Visual Complexity interaction (b < −0.01, SE = 0.01, t(58,480) = −0.01, *p* = 0.992). However, the Word Length × Word Frequency interaction was significant (b = −0.01, SE < 0.01, t(19,550) = −4.81, *p* < 0.001), indicating that the word frequency effect was more pronounced for long words than for short words (see Figure 1). The Word Length × Visual Complexity interaction was also significant (b = −0.03, SE = 0.01, t(17,790) = −2.76, *p* < 0.01), reflecting a larger visual complexity effect for short words than for long words (see Figure 2).

#### 6.4.2. Gaze Duration

Gaze duration, reflecting early lexical processing, showed significant main effects of all four fixed factors as well as two significant interactions.

Main effects. Children showed significantly longer gaze durations than adults (b = 0.26, SE < 0.01, t(40,100) = 31.63, *p* < 0.001). Long words received longer gaze durations than short words (b = 0.15, SE < 0.01, t(17,140) = 18.18, *p* < 0.001). Low-frequency words elicited longer gaze durations (b = −0.01, SE < 0.01, t(52,600) = −8.19, *p* < 0.001). High visual complexity words also produced longer gaze durations (b = 0.11, SE < 0.01, t(49,190) = 13.92, *p* < 0.001).

**Figure 3:**
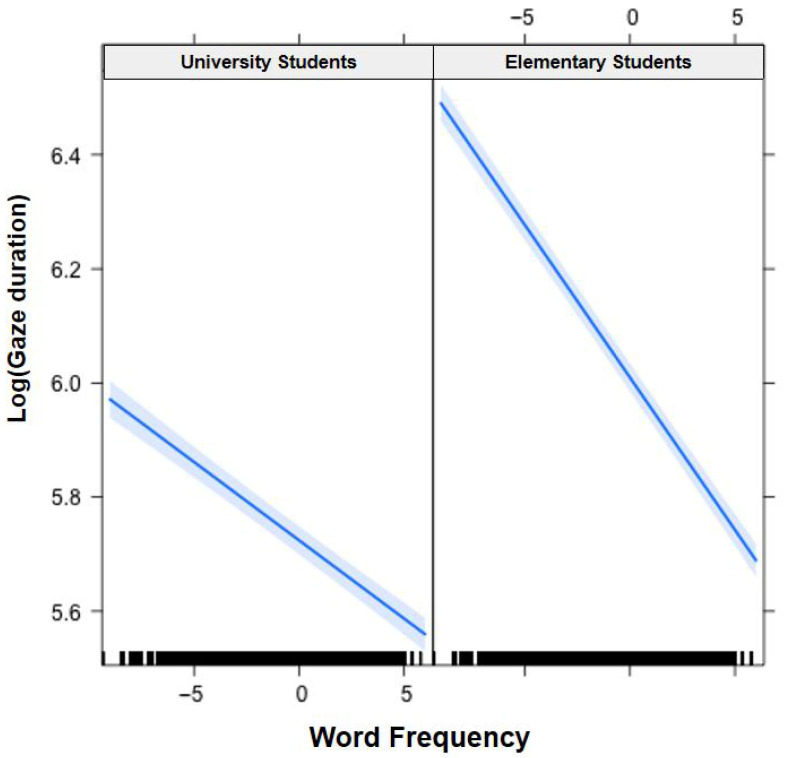
Group × Word Frequency interaction on gaze duration. Children demonstrated a larger word frequency effect than adults. Blue solid lines indicate linear regression fit lines, and light blue shaded areas represent 95% confidence intervals around the fit lines. Black ticks at the bottom are rug plots showing the distribution of observations along the x-axis.

**Figure 4:**
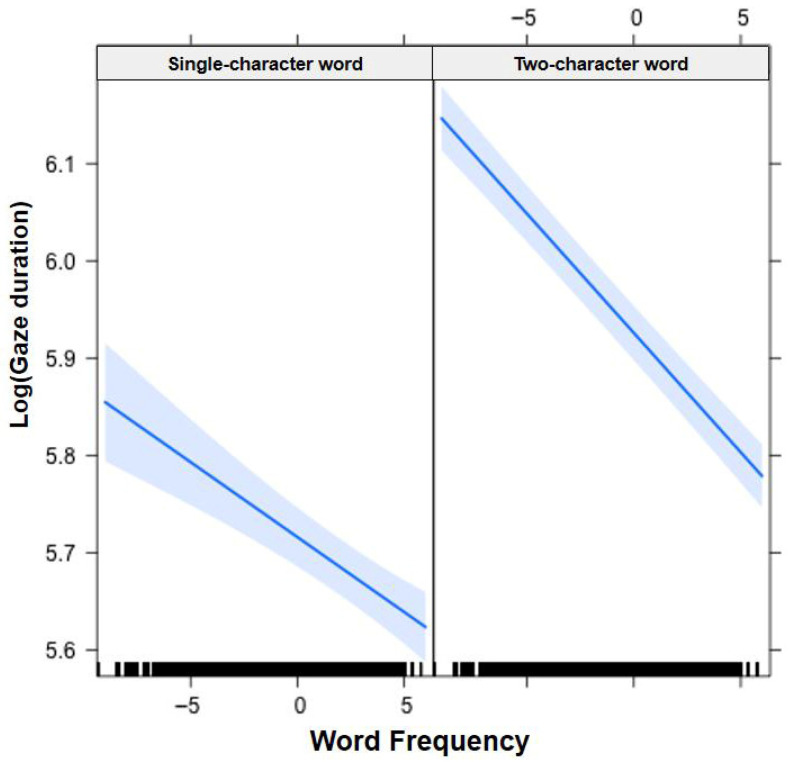
Word Length × Word Frequency interaction on gaze duration. All participants showed a larger word frequency effect for two-character (long) words than for single-character (short) words. Blue solid lines indicate linear regression fit lines, and light blue shaded areas represent 95% confidence intervals around the fit lines. Black ticks at the bottom are rug plots showing the distribution of observations along the x-axis.

Interactions. The Group × Word Frequency interaction was significant (b < −0.01, SE < 0.01, t(58,470) = −3.10, *p* < 0.01), with children showing a larger word frequency effect than adults (see Figure 3). The Group × Visual Complexity interaction was not significant (b = 0.02, SE = 0.02, t(58,500) = 1.07, *p* = 0.283). The Word Length × Word Frequency interaction was significant (b = −0.02, SE < 0.01, t(43,960) = −6.83, *p* < 0.001), with a larger frequency effect for long words (see Figure 4). The Word Length × Visual Complexity interaction did not reach significance (b = 0.02, SE = 0.02, t(42,320) = 1.33, *p* = 0.182).

#### 6.4.3. Total Fixation Duration

Total fixation duration showed significant main effects of all four factors and all four interactions.

Main effects. Children spent more time fixating target words (b = 0.47, SE < 0.01, t(42,150) = 60.53, *p* < 0.001). Long words received longer total fixation durations (b = 0.24, SE < 0.01, t(30,730) = 29.32, *p* < 0.001). Low-frequency words elicited longer durations (b = −0.02, SE < 0.01, t(56,660) = −10.43, *p* < 0.001). High visual complexity words produced longer durations (b = 0.14, SE < 0.01, t(55,110) = 17.93, *p* < 0.001).

**Figure 5:**
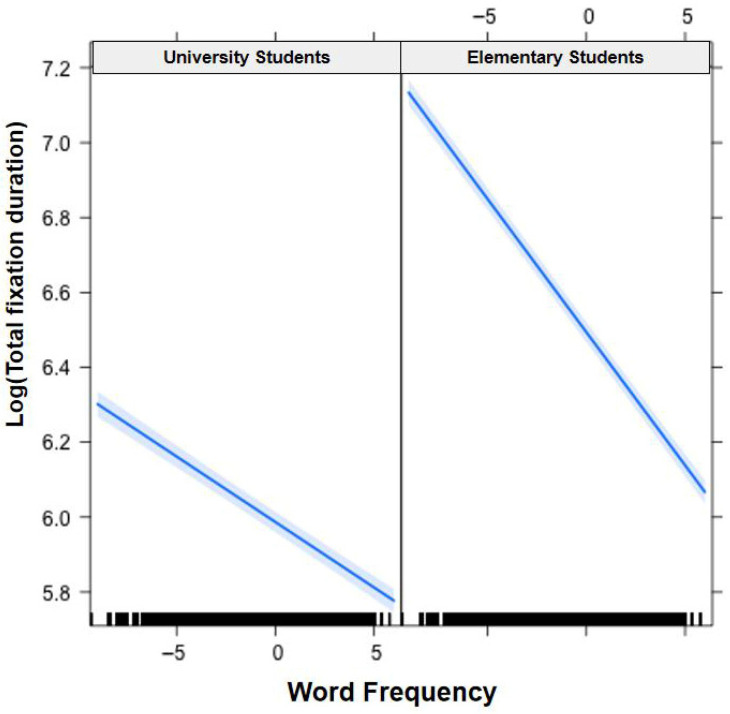
Group × Word Frequency interaction on total fixation duration. Children showed a larger word frequency effect than adults. Blue solid lines indicate linear regression fit lines, and light blue shaded areas represent 95% confidence intervals around the fit lines. Black ticks at the bottom are rug plots showing the distribution of observations along the x-axis.

**Figure 6:**
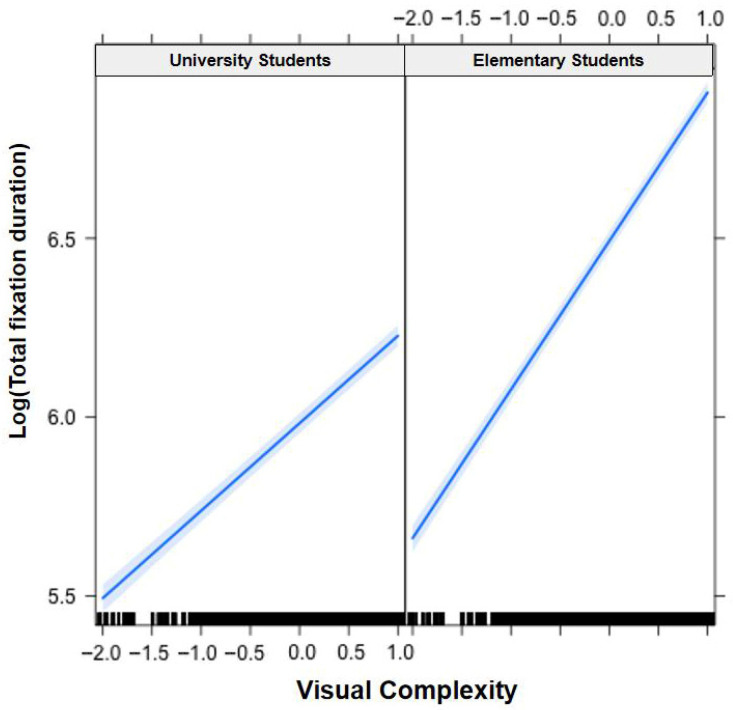
Group × Visual Complexity interaction on total fixation duration. Children showed a larger visual complexity effect than adults. Blue solid lines indicate linear regression fit lines, and light blue shaded areas represent 95% confidence intervals around the fit lines. Black ticks at the bottom are rug plots showing the distribution of observations along the x-axis.

**Figure 7:**
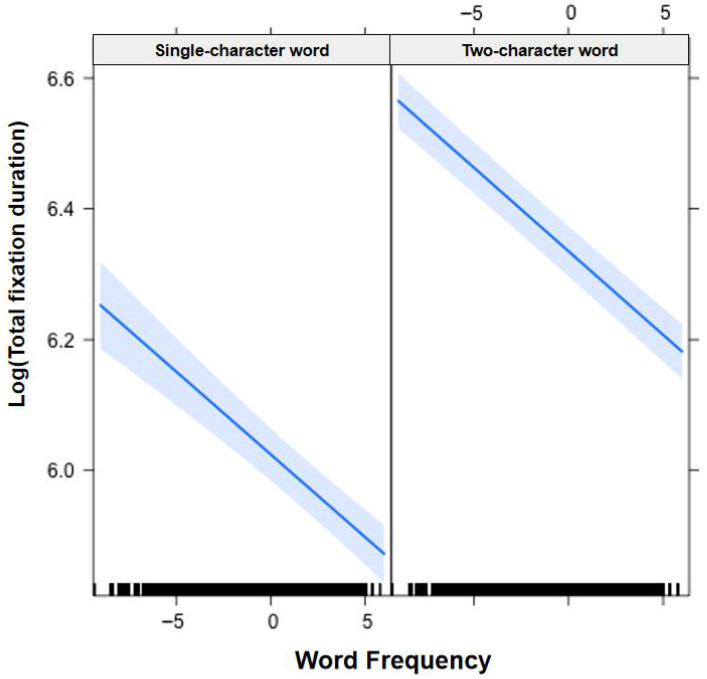
Word Length × Word Frequency interaction on total fixation duration. The magnitude of the frequency effect differed between single-character and two-character words. Blue solid lines indicate linear regression fit lines, and light blue shaded areas represent 95% confidence intervals around the fit lines. Black ticks at the bottom are rug plots showing the distribution of observations along the x-axis.

**Figure 8:**
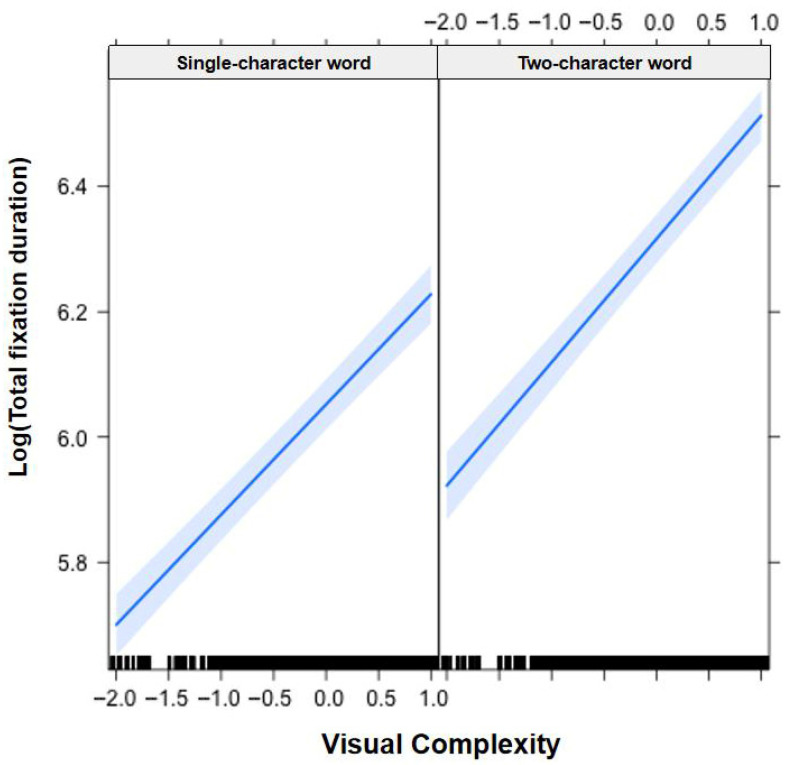
Word Length × Visual Complexity interaction on total fixation duration. The magnitude of the visual complexity effect differed between single-character and two-character words. Blue solid lines indicate linear regression fit lines, and light blue shaded areas represent 95% confidence intervals around the fit lines. Black ticks at the bottom are rug plots showing the distribution of observations along the x-axis.

Interactions. The Group × Word Frequency interaction was significant (b = −0.01, SE < 0.01, t(58,490) = −4.25, *p* < 0.001; see Figure 5). The Group × Visual Complexity interaction was significant (b = 0.05, SE = 0.01, t(58,530) = 3.44, *p* < 0.001; see Figure 6). The Word Length × Word Frequency interaction was significant (b = −0.02, SE < 0.01, t(52,830) = −5.59, *p* < 0.001; see Figure 7). The Word Length × Visual Complexity interaction was significant (b = 0.06, SE = 0.02, t(52,020) = 4.07, *p* < 0.001; see Figure 8).

#### 6.4.4. Summary of Word-Level Results

The word-level analyses revealed robust and consistent effects. First, word length exerted pervasive effects across all measures, confirming that word length is a fundamental determinant of oculomotor behaviour in unspaced Chinese text. Second, word frequency effects emerged primarily in later processing stages (gaze duration and beyond) rather than at initial fixation, suggesting that frequency modulates lexical integration rather than the earliest stages of visual recognition in oral reading. Third, visual complexity significantly affected both early and late measures, demonstrating that stroke count influences processing from initial encoding through later reanalysis. Critically, children showed disproportionately larger word frequency and visual complexity effects on total fixation duration, indicating that immature readers expend greater cognitive resources when processing low-frequency and visually complex words during oral reading. Furthermore, word length consistently moderated the effects of both word frequency and visual complexity, supporting the visual and linguistic constraints hypothesis in Chinese oral reading development.

## 7. Discussion

The present study examined how word frequency, word length, and visual complexity jointly influence eye movement behaviour during Chinese sentence oral reading, and how these effects differ between third-grade children and university adults. Using a comprehensive set of early and late eye-movement measures, the results revealed pervasive word length effects across all temporal indices, larger word frequency and visual complexity effects in children than in adults, and critically, a moderating role of word length on frequency and complexity effects, with the interaction patterns varying across processing stages. These findings advance our understanding of the multi-level architecture of lexical processing during oral reading and provide new developmental evidence for the visual and linguistic constraints hypothesis [13].

Word length emerged as the most stable and pervasive influence on eye movement behaviour. Longer words (two-character words) elicited longer first fixation durations, longer gaze durations, and greater total reading times than single-character words in both children and adults. This finding converges with recent Chinese reading research demonstrating that word length remains a fundamental determinant of oculomotor control even in the absence of interword spaces [22,23]. Theoretical frameworks such as E-Z Reader [15] and SWIFT [16] attribute the word length effect to the decline in visual acuity from the fovea to the parafovea: as word length increases, a greater proportion of constituent characters falls into regions of reduced perceptual resolution, necessitating longer fixations. The present finding that word length effects were equally robust in children and adults suggests this influence reflects a fundamental property of the visual system rather than a developmentally transient phenomenon.

Word frequency significantly modulated gaze duration and all subsequent measures, with low-frequency words eliciting longer fixations than high-frequency words [8,9,10]. Two aspects of the developmental comparison merit particular attention. First, children exhibited substantially larger frequency effects than adults, particularly on late measures such as total fixation duration. This finding is consistent with the lexical quality hypothesis and indicates that immature readers are disproportionately disadvantaged when processing low-frequency items [35]. Second, word frequency effects did not reach significance in first fixation duration for either group, emerging instead at gaze duration and beyond. This delayed emergence differs from patterns observed in silent reading, where frequency effects often appear at initial fixation [34], and is consistent with the proposal that the additional phonological demands of oral reading postpone frequency-sensitive lexical retrieval [42]. Developing readers may experience particular difficulty when processing low-frequency words during oral reading, as the dual demands of visual recognition and overt articulation place additional strain on processing resources [50,51].

Visual complexity, operationalized as stroke count, exerted significant effects on both early and late measures. High-complexity characters elicited longer first fixation durations and longer gaze durations than low-complexity characters, confirming that stroke count constitutes a genuine constraint on lexical processing [11,30,31]. The influence on first fixation duration implicates early visual pattern matching and orthographic encoding as a primary locus of complexity-driven difficulty. Importantly, the visual complexity effect was not confined to early measures but extended into total reading time, suggesting that the processing consequences of high visual complexity propagate downstream to affect phonological retrieval and lexical integration. The developmental dissociation observed here—some early processing similarities between children and adults, but with notable differences in first fixation duration and gaze duration, and greater sensitivity to visual complexity in children on late measures—suggests that developmental differences may be more pronounced in later measures, but the current design does not isolate visual encoding, lexical access, and phonological planning strongly enough to support a specific processing architecture [52].

The most theoretically significant finding concerns the moderating role of word length. Frequency effects were amplified for long (two-character) words on both early and late measures, whereas the visual complexity effect was more pronounced for short (single-character) words on early measures but showed a different pattern on some late measures. This moderating role of word length provides novel evidence for the visual and linguistic constraints hypothesis [13], although the interaction patterns differed between early and late measures. Long words possess greater linguistic content—more extensive semantic representations and more elaborate phonological structures—than short words. When a long word is additionally low in frequency, the combination of increased linguistic load and reduced representational strength creates a multiplicative processing burden. Conversely, for single-character words, the sole character constitutes the entire lexical unit; therefore, its visual properties are directly and maximally consequential for identification. For two-character words, informational redundancy across characters effectively dilutes the impact of any single character’s visual complexity. This dilution arises because visual complexity for two-character words was operationalized as the mean stroke count across constituent characters, so that extreme stroke counts of individual characters were averaged toward intermediate values, attenuating the pure stroke-count effect observed for single-character words. These cross-dimensional interactions suggest that lexical processing operates through dynamically interacting constraints rather than through isolated, modular stages [52,53].

At the theoretical level, the results of this study support a multi-level cascaded architecture of lexical processing in which visual, orthographic, phonological, and lexical information flow continuously between processing stages [53,54]. The finding that visual complexity effects persisted into late measures, that frequency effects were modulated by word length, and that children’s processing deficits were selective rather than global all converge on the conclusion that information flows continuously between levels. This architectural conception aligns more closely with connectionist and distributed-processing accounts of reading than with strictly sequential stage-based models. At the practical level, the findings carry important implications for reading instruction. The observation that children exhibited substantially larger frequency and complexity effects highlights the importance of providing beginner readers with texts containing predominantly high-frequency, low-complexity vocabulary. The finding that word length modulates both frequency and complexity effects further suggests that instructional materials should be designed with attention to the joint distribution of these lexical properties rather than to any single dimension in isolation.

### Limitations and Future Directions

Several limitations of this study should be acknowledged. First, the developmental comparison was limited to third-grade children and university adults, leaving the intermediate developmental trajectory unexplored. Future studies should include a wider age range and use longitudinal designs to clarify within-individual change. Second, the study examined only single-character and two-character words; including longer words would test whether the observed cross-dimensional moderation effects generalise to a broader range of Chinese lexical units. Third, the sample was relatively homogeneous in educational background and reading ability. Including children with reading difficulties or bilingual backgrounds would improve ecological validity. Finally, because the present study focused on oral reading, a direct comparison with silent reading using the same materials is needed to isolate the contribution of overt phonological output.

## 8. Conclusions

The present study provides a comprehensive examination of how word frequency, word length, and visual complexity interact to shape eye-movement behaviour during Chinese oral sentence reading, and how these interactions differ between children and adults. Three principal findings emerge. First, word length exerted pervasive effects across all temporal measures in both groups, confirming its status as a fundamental determinant of oculomotor control in unspaced Chinese text. Second, children exhibited larger word frequency and visual complexity effects than adults, particularly on late measures such as total fixation duration, indicating that immature readers expend disproportionate cognitive resources when processing low-frequency and visually complex lexical items during oral reading. Third, and most notably, word length functioned as a cross-dimensional moderator: frequency effects were amplified for long (two-character) words on both early and late measures, whereas the visual complexity effect was more pronounced for short (single-character) words on early measures but showed a different pattern on some late measures. This pattern offers novel support for the visual and linguistic constraints hypothesis [13] and suggests that lexical processing operates through dynamically interacting visual and linguistic constraints rather than through isolated, modular stages. The results suggest that developmental differences may be more pronounced in later measures, but the current design does not isolate visual encoding, lexical access, and phonological planning strongly enough to support a specific processing architecture. These results contribute to a more nuanced understanding of the multi-level cascaded architecture of reading, inform the development of computationally explicit models of eye movement control, and carry practical implications for the design of developmentally appropriate reading instruction and text selection for young learners.

## Figures and Tables

**Table 1:** Number of Words in Each Word Frequency and Visual Complexity Group.

	High-Frequency Words	Low-Frequency Words
High visual complexity	41	274
Low visual complexity	485	273

**Table 2:** Eye Movement Data for Adults and Children in Oral Sentence Reading.

Measure	University Students	Elementary Students
Forward Saccade Size (characters)	2.11 (0.62)	1.70 (0.53)
Regression Count	3.80 (2.12)	6.46 (3.61)
Total Reading Time (ms)	4877 (1153)	8876 (2881)

Note: Values in parentheses are standard deviations.

**Table 3:** Fixed-Effect Estimates for Sentence-Level Eye Movement Measures.

Outcome	Effect	b	SE	t	95% CI
Forward Saccade Size	Intercept	0.60	0.01	48.59 ***	0.58–0.62
Forward Saccade Size	Group	−0.22	0.02	−9.23 ***	−0.27–0.17
Regression Count	Intercept	1.43	0.02	58.99 ***	1.39–1.48
Regression Count	Group	0.53	0.05	11.21 ***	0.43–0.62
Total Reading Time	Intercept	8.76	0.01	653.68 ***	8.73–8.78
Total Reading Time	Group	0.58	0.02	24.09 ***	0.53–0.63

Note: *** *p* < 0.001.

**Table 4:** Fixed-Effect Estimates for Word-Level Eye Movement Measures.

Outcome	Effect	b	SE	t	95% CI
First Fixation Duration	Intercept	5.56	<0.01	616.18 ***	5.55–5.58
First Fixation Duration	Group	0.09	<0.01	15.00 ***	0.08–0.11
First Fixation Duration	Word Length	−0.04	<0.01	−6.28 ***	−0.05–0.03
First Fixation Duration	Word Frequency	<−0.01	<0.01	−0.97	0–0.06
First Fixation Duration	Visual Complexity	0.05	<0.01	8.40 ***	0.04–0.06
First Fixation Duration	Group × Word Frequency	<−0.01	<0.01	−0.69	−0.01–0
First Fixation Duration	Group × Visual Complexity	<−0.01	0.01	−0.01	−0.02–0.02
First Fixation Duration	Word Length × Word Frequency	−0.01	<0.01	−4.81 ***	−0.02–0.01
First Fixation Duration	Word Length × Visual Complexity	−0.03	0.01	−2.76 **	−0.06–0.01
Gaze Duration	Intercept	5.83	0.01	499.22 ***	5.80–5.90
Gaze Duration	Group	0.26	<0.01	31.63 ***	0.24–0.28
Gaze Duration	Word Length	0.15	<0.01	18.18 ***	0.13–0.17
Gaze Duration	Word Frequency	−0.01	<0.01	−8.19 ***	−0.02–0.01
Gaze Duration	Visual Complexity	0.11	<0.01	13.92 ***	0.10–0.13
Gaze Duration	Group × Word Frequency	<−0.01	<0.01	−3.10 **	−0.02–0
Gaze Duration	Group × Visual Complexity	0.02	0.02	1.07	−0.01–0.05
Gaze Duration	Word Length × Word Frequency	−0.02	<0.01	−6.83 ***	−0.03–0.02
Gaze Duration	Word Length × Visual Complexity	0.02	0.02	1.33	−0.01–0.05
Total Fixation Duration	Intercept	6.19	0.01	504.38 ***	6.16–6.21
Total Fixation Duration	Group	0.47	<0.01	60.53 ***	0.46–0.49
Total Fixation Duration	Word Length	0.24	<0.01	29.32 ***	0.22–0.25
Total Fixation Duration	Word Frequency	−0.02	<0.01	−10.43 ***	−0.02–0.01
Total Fixation Duration	Visual Complexity	0.14	<0.01	17.93 ***	0.12–0.15
Total Fixation Duration	Group × Word Frequency	−0.01	<0.01	−4.25 ***	−0.02–0
Total Fixation Duration	Group × Visual Complexity	0.05	0.01	3.44 ***	0.02–0.08
Total Fixation Duration	Word Length × Word Frequency	−0.02	<0.01	−5.59 ***	−0.02–0.01
Total Fixation Duration	Word Length × Visual Complexity	0.06	0.02	4.07 ***	0.03–0.09

Note: ** *p* < 0.01, *** *p* < 0.001.

## Data Availability

The raw data supporting the conclusions of this article will be made available by the authors on request.
